# Impact of local and systemic antimicrobials on leukocyte- and platelet rich fibrin: an in vitro study

**DOI:** 10.1038/s41598-022-06473-4

**Published:** 2022-02-17

**Authors:** S. A. M. Siawasch, C. Andrade, A. B. Castro, W. Teughels, A. Temmerman, M. Quirynen

**Affiliations:** 1 Department of Oral Health Sciences, Periodontology, KU Leuven & Dentistry, University Hospitals Leuven, Kapucijnenvoer 33, blok a - bus 07001, 3000 Leuven, Belgium; 2 Faculty of Dentistry, Postgraduate Implant Program, University of the Andes, Santiago, Chile

**Keywords:** Applied microbiology, Drug delivery, Periodontitis

## Abstract

The aim of this study was to evaluate the effect of local and systemic administration of antimicrobials to leukocyte- and platelet-rich fibrin (L-PRF). For part A, 16 tubes of venous blood were collected from each of eight systemically healthy subjects. Prior to blood centrifugation, 12 of the 16 tubes were injected with 0.125 ml, 0.25 ml or 0.50 ml metronidazole solution. One set of L-PRF membranes was used to assess the release of vascular endothelial growth factor AB, platelet-derived growth factor, transforming growth factor beta 1, and bone morphogenetic protein 2 at indicated time points. The metronidazole release over time by L-PRF membranes was also evaluated. The remaining L-PRF membranes were placed on the surface of agar plates inoculated with three different periodontal pathogens to determine their antibacterial activity. For part B, another six subjects were enrolled with three subjects taking 2 g amoxicillin and three subjects 500 mg metronidazole as prophylaxis prior to a periodontal treatment. Before and 2 h after consuming one of the prescribed antimicrobials, three tubes of blood were collected for preparing L-PRF membranes. These membranes were used to measure the antibacterial activity against periodontal pathogens. No statistically significant difference could be found in the release of growth factors between L-PRF membranes with and without incorporation of metronidazole solution. The release of metronidazole could be detected up to day 3, however with the highest concentration during the first 4 h. This concentration was dose dependent. The antibacterial capacity of L-PRF membranes increased significantly for both the systemic intake, and after the addition of metronidazole solution to the blood tubes before centrifugation, the latter again dose dependent. The antibacterial capacity of L-PRF against the periodontal pathogens tested can significantly be enhanced by the addition of antimicrobials, without disadvantage for the release of growth factors.

## Introduction

Over the past 10 years, the scientific research on platelet-rich fibrin (PRF) has tremendously increased. This resulted in more insight into the biological and mechanical properties, but also in new preparation strategies. PRF is a second generation platelet concentrate and successor of platelet-rich plasma (PRP)^1^. Biological features as fully autogenous, angiogenic activity, continuous delivery of growth factors, recruitment of active cells, antimicrobial, and analgesic activity are the main advantages of PRF^2–5^. Since PRF has more clinical advantages than PRP, it has been more widely applied in clinical practice^6–8^.

Substantial effort is paid to evaluate the release of growth factors by PRF. Most studies revealed the release of platelet-derived growth factor (PDGF), vascular endothelial growth factor (VEGF), and transforming growth factor beta (TGF-β) even after 7 days. In some studies, also the release of bone morphogenetic proteins (BMPs) could be detected^9–11^. In addition, also the antimicrobial activity of PRF has been investigated. PRF showed an inhibitory effect on the growth of the main periodontal pathogens, such as *Porphyromonas gingivalis*, *Prevotella intermedia*, and *Fusobacterium nucleatum*^12^.

Nowadays, several modifications to the original leukocyte- and platelet-rich fibrin (L-PRF) protocol have been introduced, including advanced platelet-rich fibrin (A-PRF) and advanced platelet-rich fibrin + (A-PRF +) depending on the preparation protocol^9–11,13^. For preparing L-PRF, red tubes filled with fresh blood are centrifuged at a relative centrifugal force in area where L-PRF clot is formed (RCF_clot_) of 408 g for 12 min. The centrifugation protocol for A-PRF and A-PRF + is a RCF_clot_ of 193 g for 14 min and a RCF_clot_ of 245 g for 8 min, respectively^14^. Unfortunately, the available literature does not provide clear evidence in significant clinical advantage of one above the other.

The regenerative potential of PRF allows to use it as sole filling biomaterial in different periodontal and implant therapy. Beneficial effects have been described during sinus floor elevation, alveolar ridge preservation, periodontal plastic surgery, and regeneration of intra-bony defects^15–19^. Moreover, the application of PRF in conjunction with another bio-active material has also been described in the literature. A mixture of PRF with a xenograft bone substitute can be used for guided bone regeneration procedure^20,21^. Furthermore, PRF has been combined with drugs like metformin, statin, and bisphosphonate to promote the healing capacity of PRF^22–27^. In another study, L-PRF has been modified with silver nanoparticles to improve the mechanical properties and antibacterial activity^28^.

A recent publication proposed to use L-PRF as a delivery system for antimicrobials^29^. In this article, different volumes of metronidazole, clindamycin or penicillin solutions were directly added to the blood tubes prior to centrifugation. This resulted in a significantly higher antibacterial activity against *F. nucleatum* and *Staphylococcus aureus* compared to L-PRF without antimicrobials. However, the addition of more than 0.5 ml of antimicrobial solutions negatively affected the physical properties of L-PRF membranes.

Another research group reported the same innovative approach with PRP for treating orthopaedic infections^30^. PRP clots were prepared with and without the addition of antimicrobials such as vancomycin, clindamycin, and ceftazidime. Similar to pure antimicrobials, PRP clots with antimicrobial solutions showed a significant inhibition of *S. aureus*, *Escherichia coli* and *Pseudomonas aeruginosa*. Despite that most of the antimicrobials were released after the first 10 min, a significant higher minimum inhibitory concentration (MIC) could be detected after 3 days. The release of growth factors maintained the same levels only when a low dose (1 mg) of antimicrobials was added to PRP.

As mentioned above, L-PRF and its modifications could have a beneficial effect in terms of antibacterial activity after the addition of antimicrobials. However, it is crucial to understand which concentrations of antimicrobials can provide this significant advantage. Furthermore, it is also essential to ascertain the effect of those concentrations on the biological properties of L-PRF. And last, potential adverse effects of antimicrobials to living cells in L-PRF should be taking in consideration. As determined in previous studies, L-PRF membrane contain about 50–70% of leukocytes from the initial blood sample^20,31^. Therefore, awareness should be paid to leukocyte chemotaxis and leukopenia induced by antimicrobials^32,33^.

The aim of this study was to evaluate the effect of administration of local and systemic antimicrobials on the release of growth factors and antibacterial activity by L-PRF membranes. It is hypothesized that low concentrations of antimicrobials will not affect the release of growth factors, but it will increase the antibacterial capacity of L-PRF membranes.

## Material and methods

### Population and study design

Systemically healthy volunteers were invited to participate in this study. The exclusion criteria for study enrolment were as follows: under the age of 18 years, American Society of Anesthesiology (ASA) score III or IV, use of antimicrobials in the past 6 months, use of alcohol till 3 days before the venepuncture, smoking, and pregnancy/lactation. Prior to the participation, all included subjects received information about the study purposes, and gave both verbally and written informed consent. All methods were carried out with the approval of the Ethical Committee Research UZ/KU Leuven (study number S58789, third amendment). The Helsinki Declaration as well the regulations of University Hospitals Leuven were respected. Further government guidelines regarding to COVID-19 were strictly followed.

**Figure 1:**
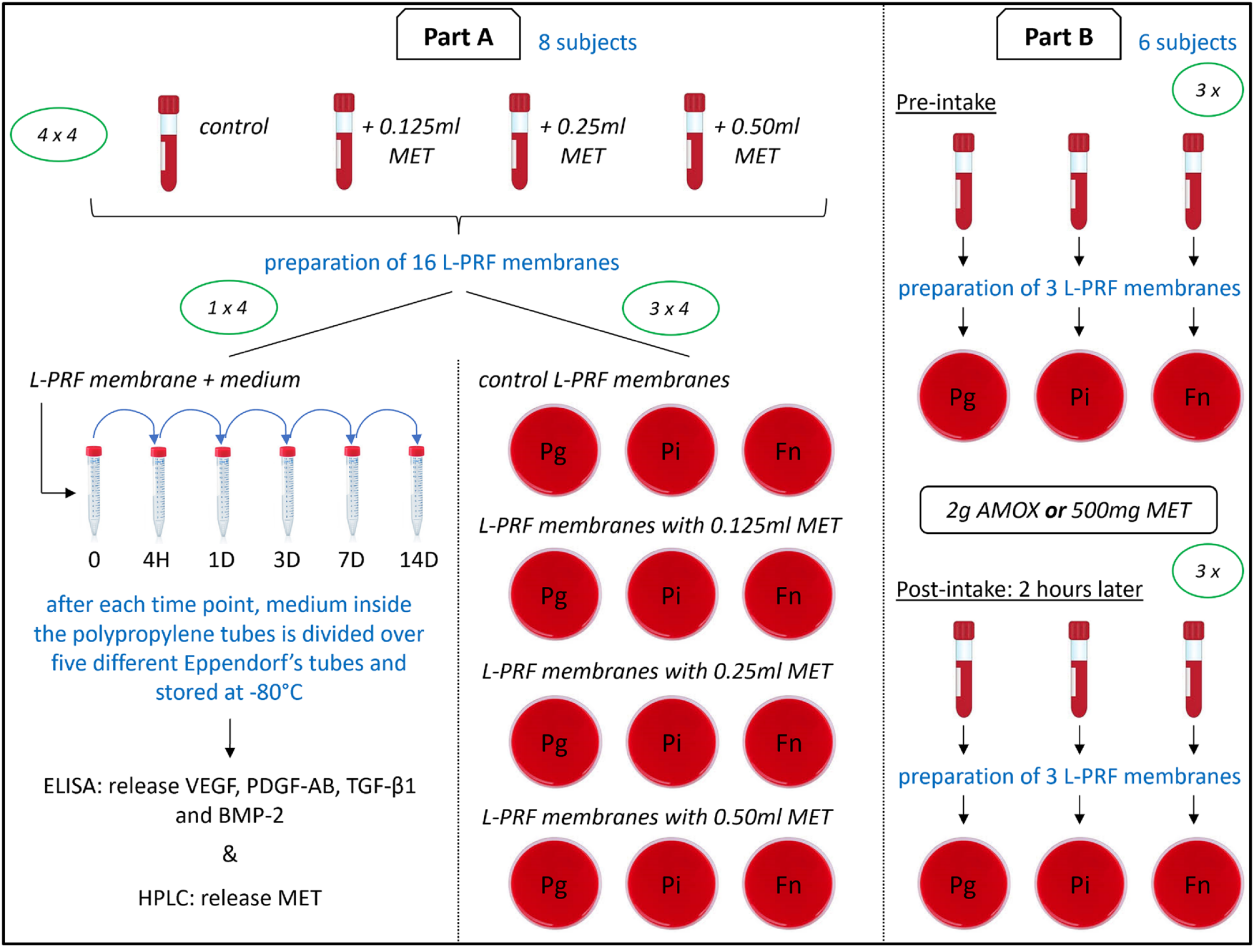
Schematic overview of the experimental setup. *MET* metronidazole, *AMOX* amoxicillin, *Pg* *Porphyromonas gingivalis, Pi* *Prevotella intermedia, Fn* *Fusobacterium nucleatum.*

The study was divided in two parts. Part A was set up to determine the effect of direct administration of local antimicrobials to the blood prior to centrifugation and part B to assess the effect of systemic antimicrobials (Fig. 1).

For part A, sixteen tubes of blood were collected from each of the eight participants to prepare L-PRF membranes. Four tubes were directly placed to the centrifuge, while in the remaining tubes 0.125, 0.25 or 0.50 ml metronidazole solution (500 mg/100 ml) [Flagyl®, Sanofi Belgium, Diegem, BE] was injected before centrifugation. The metronidazole solution was injected in the tubes with a sterile 1-ml syringe with 26-gauge needle [BD Plastipak™, Becton Dickinson, Franklin Lakes, New Jersey, USA]. So, at the end four membranes without antimicrobial (control) and twelve membranes injected with three different concentrations of metronidazole were obtained. From those sixteen membranes, four membranes (one control and one with respectively 0.125, 0.25 and 0.50 ml of metronidazole) were used to measure the release of growth factors and the release of metronidazole. The remaining twelve membranes (three controls and three with respectively 0.125, 0.25 and 0.50 ml of metronidazole) were used for determination of the antibacterial activity against periodontal pathogens *P. gingivalis*, *P. intermedia*, and *F. nucleatum*.

For part B, six patients in the need of periodontal treatment were enrolled. Each patient donated three tubes of blood before the intake of antimicrobial, and another three tubes 2 h thereafter. Three of the six patients consumed 2 g amoxicillin [Amoxicilline Sandoz®, Sandoz NV, Vilvoorde, BE] per os as endocarditis prophylaxis. Further, 500 mg metronidazole [Flagyl®, Sanofi Belgium, Diegem, BE] per os was administered as a periodontal adjunct to the other three participants. For this part of the study, only the antibacterial capacity of the L-PRF membranes was determined against the same three periodontal pathogens as in part A.

## Part A: effect of local antimicrobials

### Procedures part A

#### Preparation of L-PRF membranes

The venepuncture procedure was always performed by a trained nurse from Leuven university hospital. Intravenous blood was collected in silica-coated red cap tubes [BVBCTP-2, Intra-Spin, Intra-Lock, Florida, USA] of 9-ml by using a 21 G butterfly needle [BD Vacutainer Safety-Lok, Becton Dickinson, Franklin Lakes, New Jersey, USA]. All tubes were as soon as possible (< 1 min) centrifuged [Intraspin™, Intra-Lock, Boca Raton, Florida, USA] at RCF_clot_ 408 g for 12 min at 2700 rpm. After centrifugation the red blood cells were gently removed and the L-PRF clot was placed in a compression box [Xpression® box, Intra-Lock, Boca Raton, Florida, USA]. After 5 min of gentle compression, the exudate was squeezed out of the clot and L-PRF membranes were formed. All L-PRF membranes were weighted immediately after preparation.

#### Release of growth factors and metronidazole from L-PRF membranes

After measuring the weight, four L-PRF membranes were separately placed into a polypropylene tube of 15 ml and filled with 5 ml of Dulbecco’s Modified Eagle medium [Gibco™, Thermo Fisher Scientific, Waltham, Massachusetts, USA]. One of the L-PRF membranes was without antimicrobial and the other three with 0.125 ml, 0.25 ml, and 0.50 ml of metronidazole, respectively. The tubes were incubated at 37 °C for 4 h. Then, the L-PRF membranes were transferred to new polypropylene tubes, filled with 5 ml of the new medium, and incubated till day 1. This procedure was repeated on day 3, 7, and 14. After removing the L-PRF membranes from the tubes, the remaining medium was centrifuged [VWR™ Mega Star 6000R, VWR International BVBA, Leuven, BE] for 10 min at 1000 rpm to remove the residue. After that, the medium in each tube was distributed over 5 Eppendorf tubes of 1 ml and stored at − 80 °C. After collecting all samples from all volunteers, the release of growth factors and metronidazole was measured for all membranes at each selected time points.

*Release of growth factors from L-PRF membranes.*

Enzyme-linked immunosorbent assay (ELISA) was used to quantify the release of specific growth factors such as platelet-derived growth factor-AB (PDGF-AB), vascular endothelial growth factor (VEGF), transforming growth factor beta 1 (TGF-β_1_) [R&D Systems Europe, Abingdon, UK] and bone morphogenetic protein 2 (BMP-2) [Abbexa Ltd, Cambridge, UK]. All ELISA-tests were performed according to the manufacture guidelines and in duplicate. Detection of the ELISAs was done by a microplate reader [Varioskan™ LUX, Thermo Fisher Scientific™, Waltham, Massachusetts, USA] with measuring absorbance of 450 nm and the correction wavelength was set at 540–570 nm. For some samples, dilution was performed to adapt the concentration to the range of detection from the ELISA kits. The release of growth factors was represented in picogram per 0.1 g of L-PRF membrane, taking into account the weight of each membrane.

*Release of metronidazole from L-PRF membranes.*

To quantify the release of metronidazole, high-performance liquid chromatography (HPLC) [Agilent 1200 Series Gradient HPLC, Agilent Technologies, Santa Clara, California, USA] was performed. A volume sample of 10 µL was injected into the reversed phase column (Phenomenex Luna® C18(2), 5 µm, 100A, 250 × 4.6 mm) [Phenomenex B.V., Utrecht, NL] with the temperature maintained at 30 °C. The mobile phase was 70% 0.1 M monopotassium phosphate (KH_2_PO_4_) and 30% Acetonitrile (CH_3_CN) with a flow rate of 1 ml/min. The retention time and total runtime were 3.6 and 10 min, respectively. The ultraviolet detection wavelength was set at 318 nm and the standard calibration curve was y = 26,56 × with R^2^ = 0,9997.

#### Antibacterial capacity of L-PRF membranes

**Figure 2:**
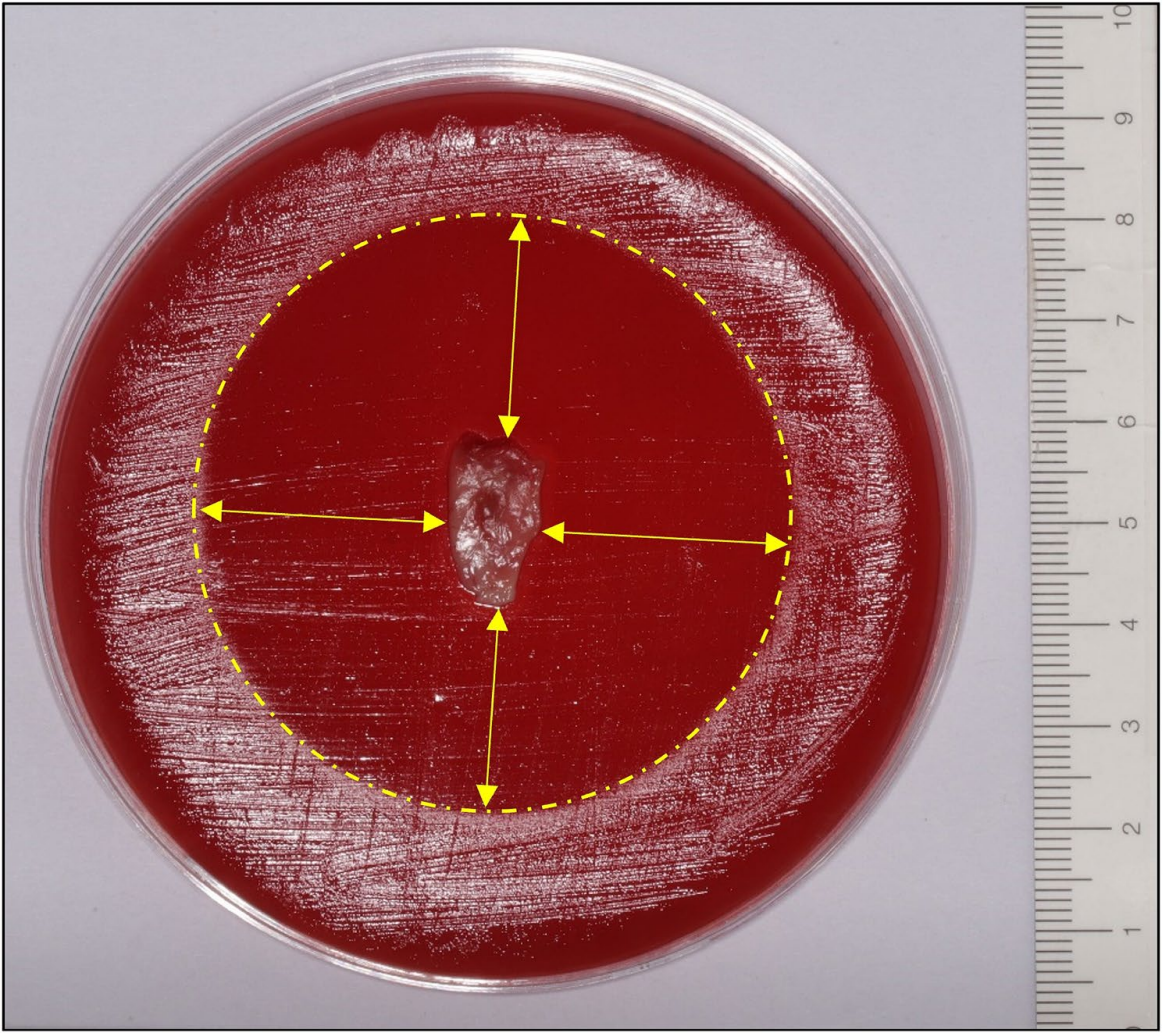
L-PRF membrane on blood agar plate after 3 days of incubation under anaerobic conditions. In this case the blood agar plate was inoculated with *Porphyromonas gingivalis* and the L-PRF membrane was prepared 2 h after consumption of 500 mg metronidazole. The circular dotted line represents the inhibition border and the arrows indicate the direction of the measurements. The ruler for calibration of the measurements is shown on the right.

The antimicrobial activity of L-PRF membranes was analysed against *P. gingivalis* (ATCC 33277), *P. intermedia* (ATCC 25611), and *F. nucleatum* (ATCC 20482) by using agar diffusion test. Those periodontal pathogens are gram-negative and anaerobic, so they could be cultured under the same conditions. An overnight culture was prepared for all bacterial strains. All three bacterial species were cultured in brain heart infusion (BHI) [Difco™, Thermo Fisher Scientific, Waltham, Massachusetts, USA] growth medium and incubated under anaerobic conditions (37 °C, 80% N_2_, 10% H_2_ and 10% CO_2_). Bacterial concentration was determined with an optical density (OD) of 500 nm by diluting the overnight culture with BHI. Two hours before the venepuncture, blood agar plates [Oxoid, Basingstoke, UK] were inoculated with 100 µl of that suspension by using a sterile cotton swab [Aptaca S.p.A., Canelli, Asti, IT]. The blood agar plates were directly incubated in anaerobic conditions. Once the L-PRF membranes were prepared, they were placed on the inoculated plates and incubated in anaerobic conditions for 3 days. Prior and 72 h after the incubation, a standardised picture of the agar plates was taken. Measurements of the inhibition distances were performed with ImageJ® version 1.53i [Image Processing and Analysis in Java, 1.8.0_172] software. Four different lines were drawn from the border of the L-PRF membrane to the border of the inhibition zone and finally the average inhibition was calculated. A fixed ruler next to the agar plates was used as a help tool to calibrate the measurements (Fig. 2).

## Part B: effect of systemic antimicrobials

### Procedures part B

The L-PRF membranes were prepared following the same protocol as for part A, but no metronidazole solution was added to any tubes. Also, the protocol for assessing the antibacterial activity with agar diffusion test was identical to part A. Unlike part A, the release of growth factors and antimicrobials was not determined.

### Statistical analysis part A and B

A weighted linear mixed model was used to assess the comparisons between the groups. Patient was the random factor and the applied weights were inversely proportional to each group's variance. To check the normality of the residuals, normal quantile plots of the residual values were used. If required, log- or square root transformation of the data were performed before analysis. When the normal quantile plots showed that the presence of below-quantification limit data disturbed the assumption of normality of data, data were considered as presence or absence of growth factor or pathogen and analysed with a generalized linear mixed model for binary data using a logit link with patient as random factor. Sidak correction was used for all comparisons between groups for simultaneous hypothesis testing. P-values less than 0.05 were considered significant and post hoc analysis was conducted. All data were analysed using S-Plus 8.0 [SolutionMetrics, Sydney, Australia] for Linux.

## Results

**Part A: effect of local antimicrobials.**

### Demographic data

Eight systemically healthy volunteers were included in part A of this study. The mean age was 37 years (SD ± 15 years). No complication was observed during blood collection.

### Release of growth factors

**Figure 3:**
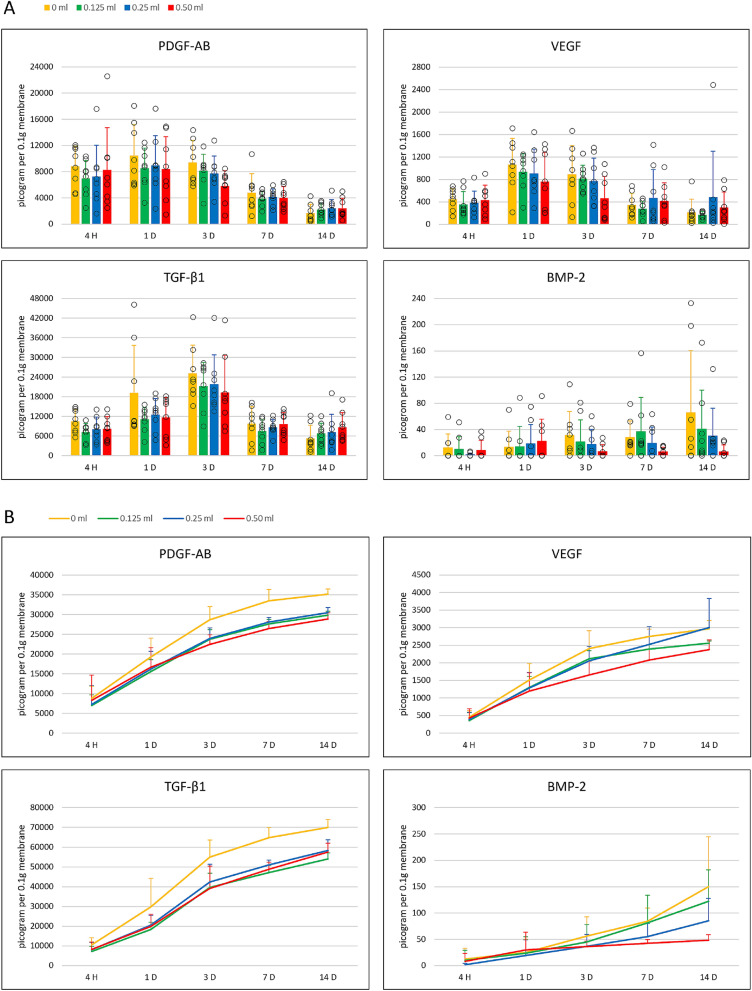
(**A**) Impact of metronidazole quantity on release of growth factors PDGF-AB, VEGF, TGF-β_1_ and BMP-2 measured at five different time points. (**B**) Impact of metronidazole quantity on cumulative release of growth factors PDGF-AB, VEGF, TGF-β_1_ and BMP-2 measured up to 14 days.

The release of growth factors PDGF-AB, VEGF, TGF-β_1_ and BMP-2 at indicated time points and as total accumulated quantities are summarized in Fig. 3A,B, respectively. In general, a similar release pattern could be observed for PDGF-AB, VEGF, and TGF-β_1_ with the most release in the first 3 time points. The release of BMP-2 was more irregular. No statistically significant differences (p > 0.05) could be detected among the four conditions: control L-PRF membranes and membranes with respectively 0.125, 0.25 and 0.50 ml metronidazole.

### Release of metronidazole

**Figure 4:**
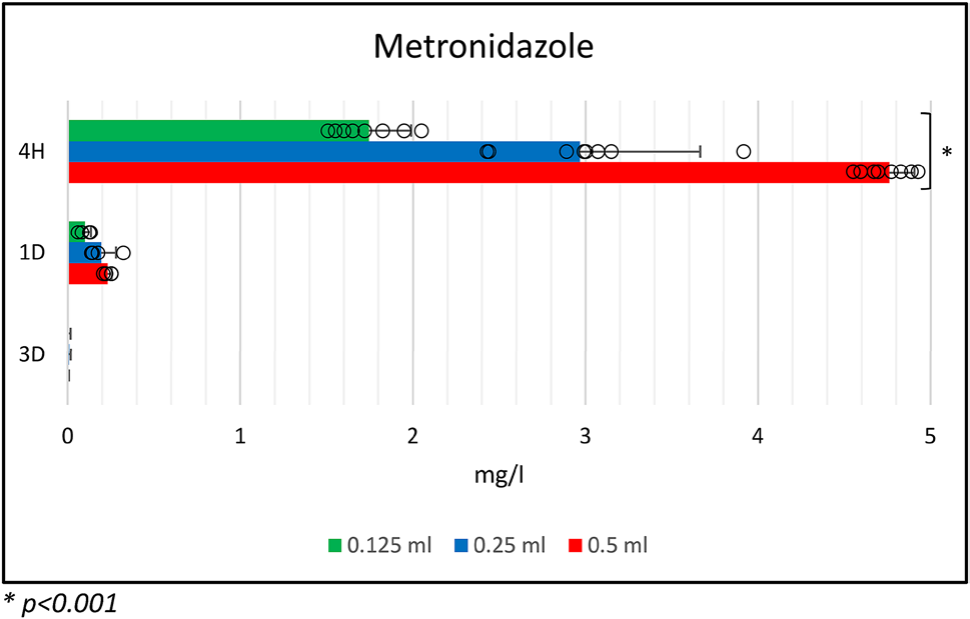
Release of metronidazole from L-PRF membranes till day 3 in relation to the injected volume of metronidazole solution.

The release of metronidazole was detected after 4 h and at day 1 and 3 (Fig. 4). The first time point showed a high release with dose dependent statistical differences (p < 0.001).

### Antibacterial capacity

The blood agar plate test revealed an inhibitory effect for all L-PRF membranes against the three periodontal pathogens (Table 1). The antibacterial effect was highest against *P. gingivalis* and lowest against *P. intermedia*. All three volumes of metronidazole contributed to a statistically significant higher inhibition distance compared to the control membranes (p < 0.01). The inhibition caused by L-PRF membranes modified with metronidazole solution was dose dependent.

**Table 1:** Impact of metronidazole solution quantity on average bacterial inhibition distances by L-PRF membranes.

	0 ml	0.125 ml	0.25 ml	0.50 ml
*Porphyromonas gingivalis*	2.00 mm^a^ SD = 0.40 mm	30.22 mm^b^ SD = 2.74 mm	31.06 mm^b^ SD = 2.79 mm	33.42 mm^c^ SD = 1.57 mm
*Prevotella intermedia*	0.48 mm^a^ SD = 0.42 mm	16.79 mm^b^ SD = 1.67 mm	20.16 mm^c^ SD = 1.42 mm	22.82 mm^d^ SD = 2.25 mm
*Fusobacterium nucleatum*	0.69 mm^a^ SD = 0.42 mm	21.21 mm^b^ SD = 3.09 mm	24.08 mm^bc^ SD = 3.99 mm	25.11 mm^c^ SD = 2.87 mm

Different letters indicate significant differences (p < 0.05) between the inhibition distances among the four conditions.

*SD* standard deviation.

**Part B: effect of systemic antimicrobials**

### Demographic data

Six systemically healthy volunteers were enrolled for this part of the study. The mean age was 49 years (SD ± 17 years). No complication was observed during blood collection.

### Antibacterial capacity

An inhibitory effect against the three periodontal pathogens could be observed for both, L-PRF membranes prepared before and after consumption of oral antimicrobials by the participants (Table 2). The membranes before antimicrobials consumption showed the highest inhibition distance against *P. gingivalis* and the lowest against *P. intermedia*. Consumption of amoxicillin resulted in significantly higher inhibition distance against all three pathogens (p < 0.01). After consumption of metronidazole a significant higher inhibition distance was found against *P. gingivalis* and *F. nucleatum*, but not against *P. intermedia*.

**Table 2:** Impact of amoxicillin and metronidazole prophylaxis on average bacterial inhibition distances by L-PRF membranes.

	AMOX-before	AMOX-after	MET-before	MET-after
*Porphyromonas gingivalis*	1.54 mm SD = 0.75 mm	19.80 mm SD = 1.72 mm	1.39 mm SD = 0.39 mm	15.68 mm SD = 0.84 mm
*Prevotella intermedia*	0.64 mm SD = 0.57 mm	15.07 mm SD = 1.42 mm	0.39 mm SD = 0.17 mm	0.87 mm SD = 0.73 mm
*Fusobacterium nucleatum*	0.90 mm SD = 0.56 mm	8.70 mm SD = 1.64 mm	0.60 mm SD = 0.38 mm	7.61 mm SD = 1.90 mm

*AMOX* amoxicillin, *MET* metronidazole, *before* prior to consumption of antimicrobial, *after* subsequent to consumption of antimicrobial, *SD* standard deviation.

## Discussion

The aim of the present study was to determine if local and/or systemic administration of antimicrobials had an impact on the growth factors release and/or antibacterial activity of L-PRF membranes. For this purpose, the most commonly used antimicrobials in periodontics, such as amoxicillin and metronidazole were investigated^34^.

The release of PDGF-AB, VEGF, TGF-β_1_, and BMP-2 were quantified at indicated time points and as total accumulated quantities. For all growth factors, a continuous release at each time point up to 14 days could be observed for both control L-PRF membranes and membranes modified with metronidazole solution. L-PRF membranes incorporated with metronidazole solution showed a similar pattern of release as control L-PRF membranes and no statistically significant differences could be detected in the amount of growth factors among the four groups.

Two studies, discussed earlier in the introduction, have already examined the addition of antimicrobials to platelet concentrates. Ten milligrams ceftazidime or 100 mg vancomycin led to a significant reduction in release of growth factors PDGF-BB and TGF-β_1_ by PRP. In addition, the administration of more than 0.5 ml antimicrobial solutions resulted in negative changes in physical properties of L-PRF^29,30^. For this reason, in the current study no volumes of metronidazole solution higher than 0.5 ml were used. This was equal to 2.5 mg (500 mg/100 ml × 0.5 ml). Administration of local or systemic antimicrobials had no impact on L-PRF clot and membrane formation.

The quantification of the release of growth factors was performed with ELISA-kits. Because no ELISA-kit is available to analyse the antimicrobials in human serum, the release of metronidazole was verified via HPLC. This is a fast, effective, and reliable technique for determination of antimicrobials^35,36^. According to previous studies, the release of antimicrobials by platelet concentrates can be detected up to 3 days if local antimicrobials are incorporated during the preparation procedure^29,30^. In the present study, the release of metronidazole by modified L-PRF membranes could also be detected up to 3 days. As expected, no release from control membranes was found. For membranes incorporated with a metronidazole solution, the release was significantly higher at the first 4 h. Nevertheless, the detected concentrations at day 1 and 3 were still far above the MIC for periodontal pathogens^37,38^.

Three different micro-organisms that have been implicated as predominant species during periodontal disease, were selected for assessing the antibacterial capacity of L-PRF membranes^39^. L-PRF membranes prepared without additional metronidazole solution (part A of the study) and prior to consumption of amoxicillin or metronidazole (part B of the study) exhibited small inhibition distances. The sequence of the highest inhibition distances was against *P. gingivalis* followed by *F. nucleatum* and finally by *P. intermedia*. This natural antibacterial activity of L-PRF membranes cohered to a previous study^12^. Modification of L-PRF membranes with 0.125, 0.25 and 0.50 ml metronidazole solution resulted in significant higher and dose dependent antibacterial activity against all three pathogens. Likewise, greater antibacterial capacity was found after systemic administration of amoxicillin and metronidazole, except for metronidazole against *P. intermedia*. Local administration of the smallest volume (0.125 ml) of metronidazole solution directly to the blood tube caused already a higher inhibition distances against all three pathogens than 2 g amoxicillin or 500 mg metronidazole as one time prophylaxis. L-PRF membranes prepared after amoxicillin prophylaxis showed a higher inhibitory effect against *P. gingivalis* and *P. intermedia* than *F. nucleatum*. That could be explained by the fact that the susceptibility of *P. gingivalis* and *P. intermedia* to amoxicillin is higher compared to *F. nucleatum*. Likewise, the absence of an inhibitory effect after systemic administration of metronidazole could be the result of a low susceptibility of *P. intermedia* to metronidazole^40^. Susceptibility of anaerobic pathogens to antimicrobials depends on the relevant species, but also on the strain and region^41^.

For part B of the study, participants donated 2 times blood, once prior and 2 h after taking antimicrobial amoxicillin or metronidazole. The second time point coincides with the mean time that takes for those drugs to reach the maximum plasma concentration (T_max_)^42^. From a technical point of view, the modification of antibacterial capacity of L-PRF seems to be easier by administrating systemic prophylaxis. However, this is less interesting when taking antimicrobial resistance into account^43^. Moreover, administration of antimicrobial solution to L-PRF tubes is not difficult, nor time-consuming, and also very simple.

This study demonstrated that addition of local antimicrobials to the L-PRF preparation procedure resulted in L-PRF membranes with significantly higher antibacterial capacity due to the release of the incorporated antimicrobial. Thus, L-PRF membranes may act as a local delivery system for antimicrobials to achieve a high concentration at infected sites, such as periodontal intra-bony defects. All drug delivery systems require a carrier that in the case of L-PRF would be the fibrin matrix. Fibrin-based matrices are well-known in tissue engineering because they contain numerous binding sites for cells, proteins, and growth factors^20^. However, previous studies reported rapid release in relative short time after antimicrobials were bound to fibrin sealants. This could be due to rapid diffusion and/or low binding affinity for specific antimicrobials^44–46^.

Incorporating L-PRF membranes with antimicrobials may bring concerns regarding global antimicrobial resistance (AMR). In part A of this study, metronidazole solution was injected directly into the blood tubes after venepuncture. In part B, six patients consumed systemic antimicrobials for medical purpose and the prescribed doses were according to the medical guidelines. So, none of the 14 subjects were unnecessary exposed to antimicrobials due to participation. With local administration of antimicrobials, a higher concentration can be reached in the intended site and less adverse effects are reported comparing to systemic use^47^. Nevertheless, local antibiotic may also contribute to AMR as has been shown in dermatology and ophthalmology^48,49^. Incorporation of L-PRF membranes with antimicrobials should not be implement as a standard protocol. It is only reasonable to add antimicrobials to L-PRF if the antibacterial activity plays a major role to achieve treatment success. This modification is a contra-indication for cases where L-PRF is only used for his regenerative and angiogenic capacity. Therefore, the clinician should always make a good consideration.

Two limitations have to be taken into consideration while processing this in vitro study. First, the small sample size with eight participants in part A and six participants in part B. The post hoc analysis revealed that in some cases a significant difference in the release of growth factors may be found with a higher sample size (Supplementary Table S1 and S2). Second, no HPLC was performed to record the release of antimicrobials in time after systemic administration. Regardless these limitations, it is worth pointing out some strengths of this study. The present study proved that antibacterial activity of L-PRF membranes can significantly be enhanced by local administration of antimicrobials to the blood tubes or by systemic prophylaxis. In addition, this study proved that greater antibacterial activity can be archived with low concentrations of antimicrobials without affecting the release of growth factors by L-PRF membranes. At last, this study explored the opportunity of L-PRF membrane as a slow-release device.

## Conclusion

Within the limitation of this study, we can conclude that administration of local and systemic antimicrobials provided L-PRF membranes with a significant greater antibacterial capacity against *P. gingivalis*, *P. intermedia*, and *F. nucleatum*. This benefit could be achieved with low concentrations of antimicrobials, which is important to maintain the biological and mechanical features of L-PRF. Now there is a need for randomized clinical trials to assess the clinical benefit of modified L-PRF membranes during periodontal and implant surgery. Possible research domains could be periodontal regenerative surgery, ridge preservation after extraction of infected tooth, and prevention of osteoradionecrosis of the jaw (ORJ) or medication-related osteonecrosis of the jaw (MRONJ).

## Supplementary Information

Supplementary Information.
