# Supplementary material for: Impact of local and systemic antimicrobials on leukocyte- and platelet rich fibrin: an in vitro study
Source: Sci Rep. 2022 Feb 17;12:2710. doi: 10.1038/s41598-022-06473-4 (PMC8854700; doi:10.1038/s41598-022-06473-4)
Supplement: Supplementary file 1 — Supplementary Information. [file 41598_2022_6473_MOESM1_ESM.docx]

Supplementary results

Post-hoc analysis was performed to find the ‘number (N) for significance’. This is a factor by which the current sample size must be multiplied to have a chance of 80% for finding a statistically significant difference. Table S1 and S2 represent the comparisons with N≤5.

Supplementary Table S1

Refers to the figure 3A in the main text: the release of growth factors measured at five different timepoints.

| **Growth factor** | **Time interval** | **Comparison** |
| --- | --- | --- |
| PDGF-AB | 4H | 0 ml vs. 0.25 ml |
|  | 1D | 0 ml vs. 0.50 ml |
|  | 3D | 0 ml vs. 0.50 ml  0.125 ml vs. 0.50 ml  0.25 ml vs. 0.50 ml |
| VEGF | 1D | 0 ml vs. 0.50 ml |
|  | 3D | 0 ml vs. 0.50 ml  0.125 ml vs. 0.50 ml  0.25 ml vs. 0.50 ml |
| TGF-β1 | 4H | 0 ml vs. 0.125 ml |
|  | 1D | 0 ml vs. 0.125 ml  0 ml vs. 0.25 ml  0 ml vs. 0.50 ml |
|  | 3D | 0 ml vs. 0.50 ml |
|  | 14D | 0 ml vs. 0.50 ml |
| BMP-2 | 4H | 0 ml vs. 0.25 ml |
|  | 3D | 0 ml vs. 0.50 ml  0.25 ml vs. 0.50 ml |
|  | 7D | 0 ml vs. 0.25 ml  0 ml vs. 0.50 ml  0.125 ml vs. 0.25 ml  0.125 ml vs. 0.50 ml |
|  | 14D | 0 ml vs. 0.50 ml  0.125 ml vs. 0.50 ml  0.25 ml vs. 0.50 ml |

Supplementary Table S2

Refers to the figure 3B in the main text: the cumulative release of growth factors measured up to 14 days.

| **Growth factor** | **Time interval** | **Comparison** |
| --- | --- | --- |
| PDGF-AB | 3D | 0 ml vs. 0.125 ml  0 ml vs. 0.25 ml  0 ml vs. 0.50 ml |
|  | 7D | 0 ml vs. 0.125 ml  0 ml vs. 0.25 ml  0 ml vs. 0.50 ml |
|  | 14D | 0 ml vs. 0.125 ml  0 ml vs. 0.25 ml  0 ml vs. 0.50 ml |
| VEGF | 1D | 0 ml vs. 0.50 ml |
|  | 3D | 0 ml vs. 0.50 ml  0.125 ml vs. 0.50 ml  0.25 vs. 0.50 ml |
|  | 7D | 0 ml vs. 0.50 ml  0.125 ml vs. 0.50 ml  0.25 vs. 0.50 ml |
|  | 14D | 0 ml vs. 0.50 ml  0.25 ml vs. 0.50 ml |
| TGF-β1 | 1D | 0 ml vs. 0.125 ml  0 ml vs. 0.25 ml  0 ml vs. 0.50 ml |
|  | 3D | 0 ml vs. 0.125 ml  0 ml vs. 0.25 ml  0 ml vs. 0.50 ml |
|  | 7D | 0 ml vs. 0.125 ml  0 ml vs. 0.25 ml  0 ml vs. 0.50 ml |
|  | 14D | 0 ml vs. 0.125 ml  0 ml vs. 0.25 ml  0 ml vs. 0.50 ml |
| BMP-2 | 4H | 0 ml vs. 0.25 ml |
|  | 1D | 0.125 ml vs. 0.50 ml |
|  | 3D | 0 ml vs. 0.125 ml  0 ml vs. 0.50 ml |
|  | 7D | 0 ml vs. 0.25 ml  0 ml vs. 0.50 ml |
|  | 14D | 0 ml vs. 0.25 ml  0 ml vs. 0.50 ml  0.125 ml vs. 0.50 ml |
